# Cutaneous eruptions associated with human polyomavirus 6 and human polyomavirus 7 in liver transplant recipients: Two case reports

**DOI:** 10.1016/j.jdcr.2026.05.045

**Published:** 2026-05-26

**Authors:** Yoni Sacknovitz, Margaret E. Scollan, Maggie H. Zhou, James Ng, Nischay Mishra, Marcus R. Pereira, Alyson N. Fox, Alejandro A. Gru, Larisa J. Geskin, Stephanie M. Gallitano

**Affiliations:** a Columbia University Vagelos College of Physicians and Surgeons, New York, New York; b Department of Dermatology, Columbia University Irving Medical Center, New York, New York; c Center for Infection and Immunity, Mailman School of Public Health, Columbia University, New York, New York; d Department of Epidemiology, Mailman School of Public Health, Columbia University, New York, New York; e Division of Infectious Diseases, Department of Medicine, Columbia University Irving Medical Center, New York, New York; f Division of Digestive and Liver Diseases, Department of Medicine, Columbia University Irving Medical Center, New York, New York; g Department of Dermatopathology, Columbia University Irving Medical Center, New York, New York

**Keywords:** immunosuppression, polyomavirus, retinoid, skin, transplant

## Introduction

Human polyomaviruses (HPyVs) are ubiquitous double-stranded DNA viruses that establish latent infections in immunocompetent hosts but can cause significant morbidity in immunocompromised patients.^1^^,^^2^

HPyV6 and HPyV7, identified in 2010, are associated with pruritic dyskeratotic dermatoses in immunosuppressed patients^3^^,^^4^; however, documented cases remain exceedingly rare.^4^^,^^5^ We report 2 polyomavirus-associated cutaneous eruptions in liver transplant recipients - 1 HPyV6, 1 HPyV7 - highlighting clinical presentation, histopathologic features, and management.

## Case reports

### Case 1: HPyV6

A 38-year-old male with Fitzpatrick skin phototype IV and alcohol-related cirrhosis underwent deceased donor liver transplantation in May 2025. The complicated post-transplant course required augmented immunosuppression. Maintenance immunosuppression included tacrolimus, mycophenolate mofetil, and prednisone.

Three months post-transplant, he developed a generalized pruritic eruption beginning on the back, spreading to face, trunk, and extremities, with concurrent fevers and diarrhea.

**Fig 1:**
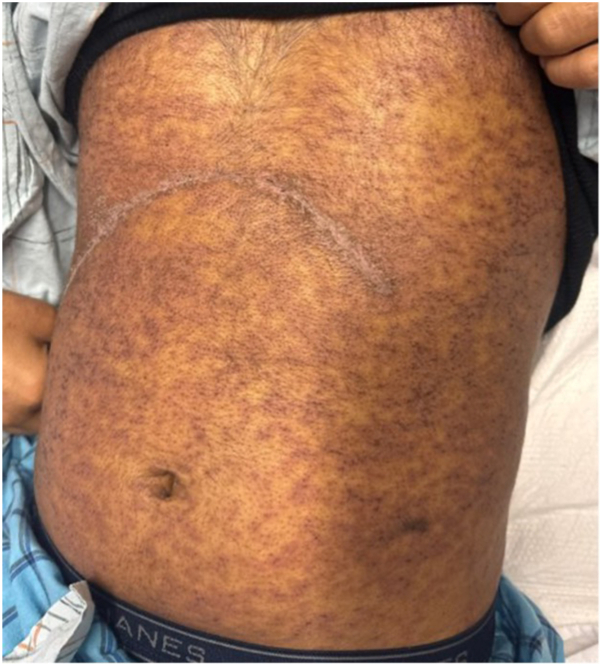
Case 1 (HPyV6): Clinical presentation at initial evaluation. Diffuse blanching pink to brown velvety papules and plaques on the trunk with scattered superficial erosions. *HPyV*, Human polyomavirus.

Examination revealed diffuse blanching pink to brown velvety papules and plaques on face, trunk, and extremities, some with superficial erosions (Fig 1). Concurrent infections included SARS-CoV-2, cytomegalovirus viremia, Salmonella gastroenteritis, and Norovirus.

**Fig 2:**
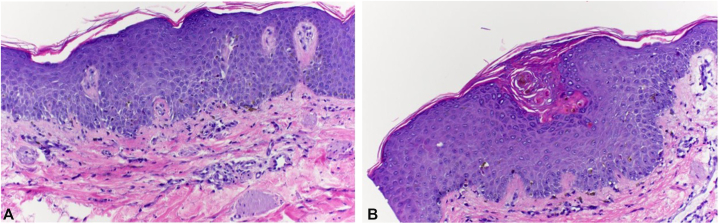
Histopathologic findings. **A,** Vacuolar alteration of the basal layer of epidermis with apoptosis, perivascular lymphoid infiltrate, scattered dyskeratotic keratinocytes, and **(B)** characteristic columns of parakeratosis (“peacock plumage” pattern). H&E stain.

Left arm punch biopsy demonstrated vacuolar alteration of the basal layer with apoptosis, perivascular lymphoid infiltrate, scattered dyskeratotic keratinocytes, and columns of parakeratosis (Fig 2, *A* and *B*), suggestive of cutaneous polyomavirus.^4^^,^^6^ VirCapSeq-VERT analysis of lesional skin confirmed HPyV6 DNA and RNA, with RNA detection indicating active viral replication.^7^

Given multiple concurrent opportunistic infections, immunosuppression was reduced: mycophenolate was held, prednisone tapered, and patient transitioned to tacrolimus monotherapy. Multidisciplinary discussion favored avoiding systemic retinoids given potential hepatotoxicity requiring close monitoring in the setting of a recent liver graft.^8^ Topical tretinoin 0.1% cream was prescribed, mixed into emollient cream (45g tretinoin in 454g jar) for nightly application to affected areas.

At 3-month follow-up, the patient reported symptomatic improvement in pruritus and no new lesions. Examination revealed thinned papules and plaques on face, scalp, shoulders, and back, with clearance of palmar lesions. He reported nightly tretinoin/emollient application to face and intermittent trunk/extremities use without irritation, remained on tacrolimus monotherapy, and was systemically well.

### Case 2: HPyV7

A 31-year-old male with Fitzpatrick skin phototype IV and primary sclerosing cholangitis underwent liver transplantation in 2012. Multiple rejection episodes required escalation to quadruple immunosuppression: prednisone, tacrolimus, mycophenolate mofetil, and everolimus.

Approximately 10 years post-transplant, he presented to dermatology with 4-months of hyperpigmented rash beginning on abdomen, spreading to trunk and upper extremities with axillary predominance, occasionally pruritic.

**Fig 3:**
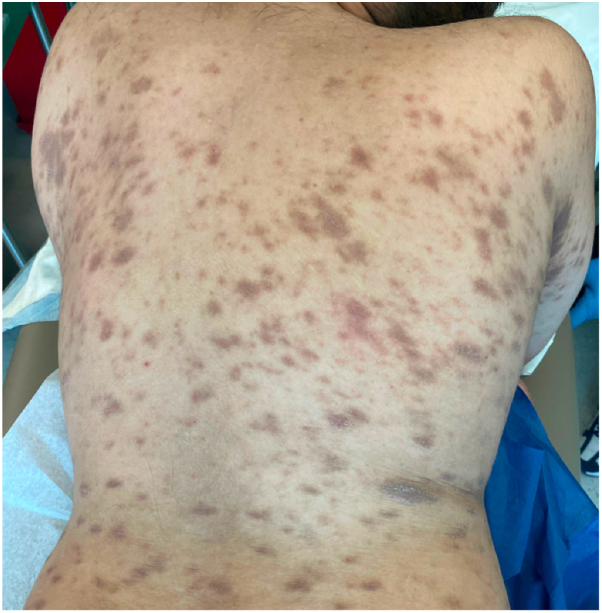
Case 2 (HPyV7): Clinical photographs. Initial presentation: scaling violaceous macules and patches with velvety appearance on the back. *HPyV*, Human polyomavirus.

Examination revealed scaly violaceous macules and thin velvety plaques on back, chest, abdomen, flanks, and bilateral axillae (Fig 3).

Right abdomen and upper back shave biopsies demonstrated plump eosinophilic keratinocytes with viral inclusion bodies at different epidermal levels, clusters of nucleated keratinocytes within stratum corneum, and mild perivascular mononuclear infiltrate. Tissue polymerase chain reaction confirmed HPyV7.

After multidisciplinary discussion, acitretin 10 mg daily was initiated with weekly liver function monitoring. Mycophenolate was reduced.

At 6 weeks on acitretin, the patient reported improvement in rash and pruritus with stable liver function tests. Mycophenolate was subsequently discontinued.

At 26-month follow-up, examination showed hyperpigmented macules on extremities and trunk consistent with postinflammatory hyperpigmentation, asymptomatic. He continues acitretin 10 mg daily with stable liver function tests; immunosuppression includes prednisone, tacrolimus, and everolimus.

## Discussion

HPyV6- and HPyV7-associated dermatoses were formally characterized by Nguyen et al in 2017, primarily affecting solid organ transplant recipients.^4^ Despite increasing recognition, documented cases remain exceedingly rare, making each report valuable. We present 2 additional cases in liver transplant recipients.

Patients with HPyV6 and HPyV7 present with velvety brown-pink thin plaques originating on trunk, spreading to extremities and face. Histologically, both share characteristic findings including dyskeratotic keratinocytes with columns of parakeratosis (“columnar dyskeratosis,” “tiered parakeratosis with dyskeratosis,” or “peacock plumage”).^4^^,^^6^ HPyV6, HPyV7, and HPyV9 are histologically indistinguishable; molecular diagnosis via quantitative polymerase chain reaction or viral sequencing is required for definitive identification.

Treatment remains challenging due to rarity and limited evidence. Immunosuppression reduction is the primary intervention. Case 1 transitioned to tacrolimus monotherapy with mycophenolate discontinuation and prednisone taper; Case 2 had mycophenolate reduced and eventually discontinued. In Case 1, immunosuppression reduction likely drove improvement, occurring concurrent with resolution of multiple infections.

Retinoids have shown efficacy when used at our institution. Retinoids promote keratinocyte differentiation and epidermal turnover, which may facilitate desquamation of virus-infected keratinocytes.^9^ Canavan et al reported complete HPyV7 remission with oral acitretin in a heart transplant recipient, and Kwan et al similarly reported complete clearance of HPyV7-associated eruption with 25 mg of oral acitretin in a kidney and pancreas transplant recipient at 3 months.^5^^,^^8^^,^^10^

In Case 2, the temporal relationship suggests acitretin played a significant role: the eruption worsened initially despite immunosuppression reduction, then improved gradually over 26 months on acitretin 10 mg daily, achieving near-complete resolution with only residual postinflammatory hyperpigmentation. In Case 1, systemic retinoids were contraindicated due to hepatotoxicity concerns in the setting of a recent liver graft; topical tretinoin 0.1% mixed with emollient was employed as an adjunctive measure, with cutaneous improvement at 3-month follow-up. For patients tolerating systemic therapy, low-dose acitretin with close hepatic monitoring appears reasonable, as demonstrated by Case 2's sustained improvement over more than 2 years. For those with contraindications—such as recent liver transplant recipients—topical tretinoin offers a safe adjunctive option.

An interesting distinction is presentation timing relative to transplant. Case 1 presented acutely at 3 months post-transplant with concurrent infections (SARS-COV-2, cytomegalovirus, Salmonella, Norovirus), suggesting high net immunosuppression. Case 2 presented approximately 10 years post-transplant without concurrent infections but with multiple rejection episodes requiring intensified immunosuppression. This suggests polyomavirus-associated dermatoses occur across transplant chronicity when immune state permits viral reactivation.

Limitations include two-case design and concurrent immunosuppression reduction in both patients, confounding retinoid attribution. Spontaneous resolution cannot be excluded. Nevertheless, these cases contribute to sparse literature on HPyV6 and HPyV7 cutaneous eruptions and provide practical guidance for clinicians. Larger studies are needed to establish optimal treatment approaches.

## Conflicts of interest

Dr Geskin has served as an investigator for J&J, Mallinckrodt, Kyowa Kirin, Soligenix, Innate, Incyte, Trillium, Merck, BMS, and Stratpharma; on the scientific advisory board for SciTech and Citius. Dr Gallitano has served on the advisory board for Priovant. Drs Gru, Fox, Pereira, Mishra, Scollan, and Authors Sacknovitz and Zhou have no conflicts of interest to declare.

## References

[1] DeCaprio J.A., Garcea R.L. (2013). A cornucopia of human polyomaviruses. Nat Rev Microbiol.

[2] Kazem S., van der Meijden E., Feltkamp M.C.W. (2013). The trichodysplasia spinulosa-associated polyomavirus: virological background and clinical implications. APMIS.

[3] Schowalter R.M., Pastrana D.V., Pumphrey K.A., Moyer A.L., Buck C.B. (2010). Merkel cell polyomavirus and two novel polyomaviruses are chronically shed from human skin. Cell Host Microbe.

[4] Nguyen K.D., Lee E.E., Yue Y. (2017). Human polyomavirus 6 and 7 are associated with a pruritic and dyskeratotic dermatosis. J Am Acad Dermatol.

[5] Bartley B.R., Moore S.A., Doan H.Q., Rady P.L., Tyring S.K. (2023). Current treatments and emerging therapies of human polyomavirus-associated skin diseases: a comprehensive review. Int J Dermatol.

[6] Ho J., Jedrych J.J., Feng H. (2015). Human polyomavirus 7-associated pruritic rash and viremia in transplant recipients. J Infect Dis.

[7] Mishra N., Ng J., Strom M.A. (2022). Human polyomavirus 9-An emerging cutaneous and pulmonary pathogen in solid organ transplant recipients. JAMA Dermatol.

[8] Canavan T.N., Baddley J.W., Pavlidakey P., Tallaj J.A., Elewski B.E. (2018). Human polyomavirus-7-associated eruption successfully treated with acitretin. Am J Transplant.

[9] Balado-Simó P., Morgado-Carrasco D., Gómez-Armayones S. (2025). An updated review of topical tretinoin in dermatology: from acne and photoaging to skin cancer. J Clin Med.

[10] Kwan K., Sears S., Callen J. (2021). Keratotic spines in a patient with pruritic and dyskeratotic dermatosis: a new clinical finding. JAAD Case Rep.

